# Anticholinergic Load Is Associated with Swallowing Dysfunction in Convalescent Older Patients after a Stroke

**DOI:** 10.3390/nu14102121

**Published:** 2022-05-19

**Authors:** Eiji Kose, Toshiyuki Hirai, Toshiichi Seki, Michiyo Okudaira, Nobuhiro Yasuno

**Affiliations:** 1 Department of Pharmacy, Teikyo University School of Medicine University Hospital, Tokyo 173-8606, Japan; yasuno@med.teikyo-u.ac.jp; 2 Department of Pharmacy, Hitachinaka General Hospital, Ibaraki 312-0057, Japan; toshiyki.hirai.dq@hitachi.com (T.H.); toshiichi.seki.cq@hitachi.com (T.S.); 3 Laboratory of Hospital Pharmacy, School of Pharmacy, Teikyo University, Tokyo 173-8606, Japan; okudaira.michiyo.si@teikyo-u.ac.jp

**Keywords:** swallowing dysfunction, anticholinergic load, functional oral intake scale, stroke rehabilitation

## Abstract

This study aimed to establish whether anticholinergic load affects the swallowing function of geriatric stroke patients in convalescent stages, as no proven association between the anticholinergic load-based Anticholinergic Risk Scale and the swallowing dysfunction in Japanese patients was known. A retrospective cohort study was conducted on hospitalized older patients undergoing rehabilitation after stroke. The study outcomes included evaluating the patients at hospital discharge using the Functional Oral Intake Scale. To evaluate the effects of an increased anticholinergic load, we used a multivariate analysis to examine whether the change in the Anticholinergic Risk Scale during hospitalization was associated with the outcome. Of 542 enrolled patients, 345 (63.7%) presented with cerebral infarction, 148 (27.3%) with intracerebral hemorrhage, and 49 (9%) with subarachnoid hemorrhage. The change in the Anticholinergic Risk Scale was independently associated with the Functional Oral Intake Scale (β = −0.118, *p* = 0.0164) at discharge. Among anticholinergics, the use of chlorpromazine, hydroxyzine, haloperidol, metoclopramide, risperidone, etc., increased significantly from admission to discharge. An increased anticholinergic load was associated with swallowing dysfunction in older patients undergoing stroke rehabilitation.

## 1. Introduction

Stroke patients exhibit a high rate of dysphagia [1], and these dysphagic patients demonstrate higher rates of mortality [2], pneumonia [1], and undernutrition [3], compared with nondysphagic ones. As is evident from the above, improving this function through indirect and direct swallowing training in stroke patients is essential. Note that a significant number of patients remain with residual dysphagia after acute care. Precedent studies revealed that 5.1% of patients admitted to convalescent rehabilitation wards in Japan come with nasogastric tubes and 1.4% even with gastrostomy [4]. To help these patients gain oral intake, providing ingestion and swallowing training in the convalescent rehabilitation ward [5] is essential, a system unique to Japan.

Researchers reported several causes of dysphagia, including cerebrovascular and neurodegenerative diseases, as well as undernutrition [6,7], aging [7], and sarcopenia [8,9]; however, drug-induced causes are also relevant [10]. Among the latter, the effects of anticholinergic drugs cannot be ignored, as anticholinergic drugs decrease salivary secretion and induce thirst, which can cause dysphagia in older patients with reduced cough reflex. Furthermore, anticholinergic drugs increase the risk of undernutrition [11]. As undernutrition progresses, muscle mass and the strength of muscles associated with swallowing decrease, resulting in impaired swallowing function. Anticholinergic drugs are prescribed to many older patients [12]. Older adults are especially susceptible to the adverse effects of these medications for various reasons, including polypharmacy, increased blood–brain barrier permeability, and pharmacokinetic and pharmacodynamic changes. Furthermore, the physiological reduction of acetylcholine reserve and the increased risk of neurodegenerative diseases involving cholinergic neurons make older people more prone to adverse effects of anticholinergic medications [13]. Therefore, when older patients are admitted to convalescent rehabilitation wards, the danger of dysphagia caused by anticholinergic drugs should be kept in mind.

For anticholinergics, the total anticholinergic load of the drugs taken is important. An index that rates each drug according to the strength of its anticholinergic effect, called the anticholinergic risk scale (ARS) score, has been established [14]. ARS is the cumulative effects of using one or more drugs with anticholinergic activity. Although associations with clinical outcomes such as hospitalization and fractures have already been reported using it [15,16,17], no studies examined its association with dysphagia yet. A previous report showed that the Anticholinergic Cognitive Burden (ACB) score was used to assess the overall burden of anticholinergic effects and examined its relationship to muscle strength. Higher ACB scores were associated with greater muscle weakness. However, this study assessed muscle strength in terms of hand-grip strength and did not address swallowing-related muscles [18]. In the acute phase of stroke, patients are often prescribed anticholinergics such as sleep medications, antipsychotics, and antidepressants to prevent restlessness, delirium, and depression. Many patients continue to take these medications after transitioning to the recovery phase. For this reason, it is crucial to evaluate anticholinergic activity.

Motivated by the current situation in the medical literature, this study evaluates the strength of anticholinergic effects using the ARS score and examines its association with dysphagia in convalescent post-stroke patients.

## 2. Materials and Methods

### 2.1. Study Design and Participants

A retrospective cohort study was conducted on 1490 participants discharged from the convalescence rehabilitation ward at Hitachinaka General Hospital between July 2010 and October 2018. Participants who were <65 years of age, nonstroke, participants with a Functional Oral Intake Scale (FOIS) score of 1 point, transferred to another hospital for treatment of comorbidities, who died in our hospital, or presented with missing data were excluded. The study subjects were categorized according to the presence or absence of an increased anticholinergic load from admission to discharge into the “ARS score (+)” and “ARS score (−)”, respectively.

### 2.2. Data Collection

Data regarding the participants’ basic information were collected from their medical records at admission and discharge. This included their age, gender, length of stay, body weight (BW), body mass index (BMI), primary diagnosis, comorbidities, number of drugs prescribed, Functional Independence Measure (FIM), and FOIS. Each dataset was based on the data at admission. The changes in the ARS and FOIS scores were calculated by subtracting their values at discharge from their counterparts at admission. Laboratory data were also collected from medical records at admission. These included Geriatric Nutritional Risk Index (GNRI) levels [19] and C-reactive protein (CRP) levels.

### 2.3. ADL Measurements

The present study used FIM as the activities of daily living (ADL) measurement. The FIM score is one of the most common measures of ADL. It includes 13 lower-order items regarding the motor function (FIM-M) and five lower-order items regarding the cognitive function (FIM-C). Each item is scored on a scale of 1 point (total assistance) to 7 points (complete independence). Taken together, the FIM total (FIM–T) score ranges from 18 to 126 points [20]. FIM scores were assessed by the multidisciplinary rehabilitation team including a rehabilitation physician, a registered dietitian, a nurse, a physical therapist, an occupational therapist, a speech–language–hearing therapist, and a pharmacist, at admission. Based on clinical observations, suitable rehabilitation was offered to all participants, regardless of their FIM score, stroke severity, or length of stay.

### 2.4. Outcome

The primary outcome of the study was the FOIS score at discharge and the change in FOIS score, which evaluates the dysphagia in patients using a 7-point (range 1–7) scale, where the highest score indicates normal swallowing ability, and the lowest score the most severe impairment [21]. The FOIS score focuses on the feeding route and food texture of a patient’s daily diet, both of which are easy to obtain as part of a dietitian’s assessment. Moreover, dietitians recorded the FOIS score at admission and discharge based on the diet served in the previous three meals.

### 2.5. Statistical Analysis

All statistical analyses were carried out using JMP Pro (Version 15, SAS Institute, Cary, NC, USA). The numerical data were checked for normality with the Shapiro–Wilk W-test. The not-normally distributed data were described by their median (interquartile range 25th–75th percentiles). A *p*-value < 0.05 was considered statistically significant. The Mann–Whitney U-test and the Chi-square test were used to analyze the differences between groups. The Kruskal–Wallis test was used for multigroup comparisons. For multiple comparison tests, the Steel–Dwass test was used. A multiple linear regression analysis was used to examine whether FOIS at discharge was independently associated with an increased ARS score. This included some adjustments for covariates including age, gender, CRP, GNRI, Alzheimer’s disease, Parkinson’s disease, BMI, change in ARS score, cerebral infarction, intracerebral hemorrhage, subarachnoid hemorrhage, FIM–T at admission, or the number of drugs prescribed at admission. Multicollinearity was assessed using the variance inflation factor coefficient. The McNemar test was used to compare the anticholinergic drugs at admission and discharge.

### 2.6. Institutional Review Board Statement

The present study was carried out following the Declaration of Helsinki, and it was approved by the ethics committee of Hitachinaka General Hospital (Date approved: 27 August 2015).

## 3. Results

### 3.1. Descriptive and Univariate Analyses

**Figure 1:**
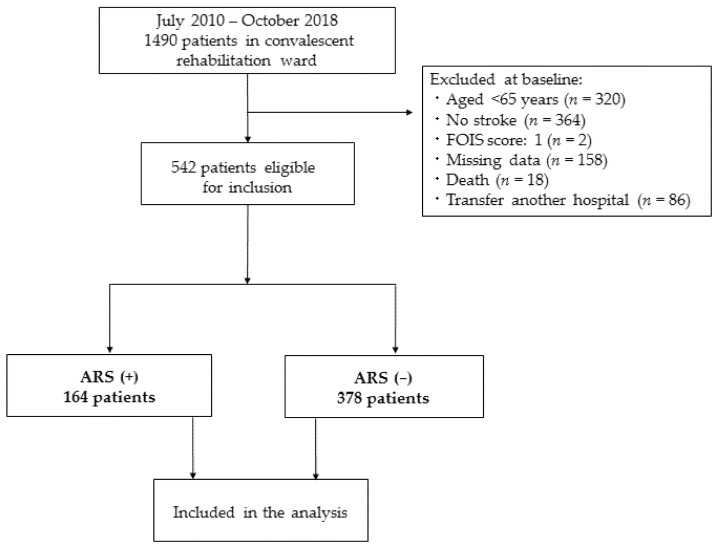
Flowchart of participant screening, inclusion criteria, and follow-up.

We enrolled 1490 participants during the study period, but it excluded 320 of them aged <65 years, 364 nonstroke participants, 2 with a FOIS score of 1 point, 158 with missing complete data, 18 patients who died in our hospital, and 86 patients who were transferred to another hospital for treatment of comorbidities. The main reason for the large number of missing data was that the outcome data, FOIS data, were missing at either or both admission or discharge. This distribution of the considered cohort is shown in Figure 1. Consequently, 542 participants with a median age of 79 years (interquartile range 73–85 years; 201 males) were included in the study, of whom 345 (63.7%) presented with cerebral infarction, 148 (27.3%) presented with intracerebral hemorrhage, and 49 (9%) presented with subarachnoid hemorrhage.

Table 1 shows the demographic characteristics by the presence (*n* = 164) and absence (*n* = 380) of an increased ARS score during hospitalization. No significant differences were found between the two groups in gender, length of stay, BW at admission, BMI at admission, primary diagnosis, comorbidities (cardiac disease, diabetes mellitus, hypertension, and Alzheimer’s disease), FOIS score, and GNRI. Conversely, age, comorbidities (Parkinson’s disease), the number of drugs prescribed at admission, FIM–T, FIM-M, FIM-C, and CRP were significantly different between the two groups.

Table 2 shows the results of the univariate analysis of outcomes. FOIS at discharge and change in FOIS score were significantly higher in the ARS (−) group compared to the ARS (+) group.

### 3.2. Multiple Linear Regression Analysis

We performed a multiple linear regression analysis for FOIS at discharge, including age, gender, CRP, GNRI, Alzheimer’s disease, Parkinson’s disease, BMI, change in the ARS score, cerebral infarction, intracerebral hemorrhage, subarachnoid hemorrhage, FIM–T at admission, and the number of drugs prescribed at admission as covariates. The analysis showed that BMI, the change in the ARS score, and FIM–T at admission were independently associated with FOIS at discharge (FOIS at discharge = 0.052 × BMI − 0.118 × change in the ARS score + 0.018 × FIM–T score + 4.258; R^2^ = 0.146, *p* < 0.001) (Table 3).

### 3.3. The Ratio of Anticholinergic Prescriptions at Admission and Discharge

**Figure 2:**
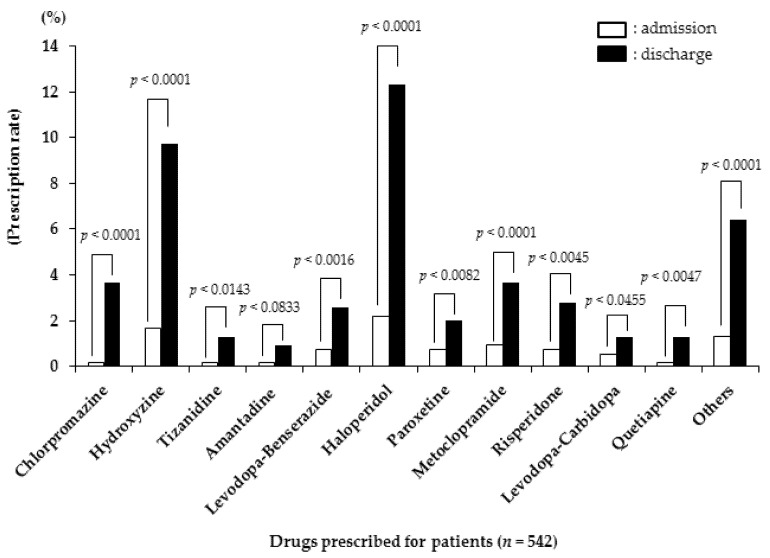
Comparison of the ratio for anticholinergic prescriptions between admission and discharge.

The comparison of anticholinergic prescriptions at admission and discharge in the 544 eligible patients is shown in Figure 2. Chlorpromazine, hydroxyzine, tizanidine, amantadine, levodopa–benserazide, haloperidol, paroxetine, metoclopramide, risperidone, levodopa–carbidopa, and quetiapine use increased significantly over the same period.

### 3.4. Subgroup Analysis of FOIS at Discharge

Subgroup analysis of FOIS at discharge was performed for gender, stroke subtype, and change in the ARS score. There were significant differences in the ARS score changes between categories, i.e., significant differences were found between the “0 point” group and “1 point increase” group and between the “0 point” group and “2 points or more increase” group. On the contrary, no significant differences were found between the categories for gender and stroke subtype (Table 4).

## 4. Discussion

The most important achievement of this study is in the finding that the amount of change in the ARS score during hospitalization was associated with the FOIS score at discharge. In other words, an increased anticholinergic load during hospitalization exhibited an independent negative effect on the swallowing function at discharge. The subgroup analysis results stated that anticholinergic load had a significant effect on swallowing function with an increase of two points or more. Another gain of this study is the realization that from admission to discharge, a marked increase was found in prescriptions for chlorpromazine and hydroxyzine, which exhibit an ARS score of three, and haloperidol, which exhibits an ARS score of one. Thus, it became clear that the concomitant use of drugs with greater anticholinergic effects, or even of ones with smaller anticholinergic effects used in combination, increases their anticholinergic effects and influences the decline in the swallowing function.

The anticholinergic load during hospitalization was independently negatively associated with the decline in the swallowing function at discharge. Anticholinergic drugs are known to affect the central nervous system, causing cognitive decline and delirium [22]. A recent systematic review and meta-analysis reported that anticholinergic drug loading in cognitively intact older adults increases the risk of cognitive decline and the development of dementia [23]. Additionally, a previous study also reported that the anticholinergic load exposure limited functional recovery in the cognitive items of ADLs “comprehension” and “memory” [24,25]. For this reason, the possibility exists that the anticholinergic load caused a drug-induced cognitive decline, resulting in decreased awareness or motivation to eat and decreased attention to feeding and swallowing, which may have affected the ability to swallow. In addition to age-related cognitive decline, the effect of drugs on cognitive function should be considered for treating dysphagia in the older patients.

Anticholinergics can cause xerostomia due to decreased saliva secretion, which can impair swallowing function. In this study, the ARS score increased from admission to discharge, in particular with 12.7% for drugs with a score of three points or higher. Chlorpromazine and hydroxyzine, with an ARS score of three points, significantly increased from admission to discharge by ~3.5% and 8.1%, respectively; haloperidol and metoclopramide, with an ARS score of one point, also significantly increased by approximately 10.1 and 2.8%, respectively. Ingestion can be divided into five stages in sending the food mass from the oral cavity through the pharynx and esophagus to the stomach. These stages are classified into: “the anticipatory stage”, when food-related information is recognized; “the preparatory stage”, when the food in the oral cavity is masticated and made into a state ready for swallowing; “the oral stage”, when the masticated food bolus is sent from the oral cavity to the pharynx; “the pharyngeal stage”, when the food bolus passes through the pharynx by the swallowing reflex, and “the esophageal stage” when the food bolus is sent into the stomach by peristalsis in the esophagus. Anticholinergics affect the “preparatory stage” of these processes. In other words, saliva secretion is reduced, making adequately forming a food mass impossible. Accordingly, for patients with dysphagia, it is necessary to select drugs by considering the strength of their anticholinergic effects as well as to provide remedies for oral dryness by administering saliva stimulants and artificial saliva and by providing oral care. Furthermore, these drugs exhibit and decrease “substance P,” a neurotransmitter distributed in the pharynx and larynx [26]. This inhibits the swallowing reflex, resulting in impaired swallowing function. Moreover, these drugs can cause extrapyramidal disorders [27]. When extrapyramidal disorders cause impaired opening and closing of the mouth and tongue movement, not only is chewing and food mass formation difficult, but delivering the food mass into the pharynx is also difficult. In this study, antipsychotics were administered primarily for disorientation and delirium. For patients with dysphagia who are taking antipsychotic medications, dosage and medication changes should be considered based on the symptoms of disorientation and delirium.

Swallowing rehabilitation generally improves the patient’s ingestion and swallowing ability, and the patient often gets well following this rehabilitation. However, some cases exist in which the ability to swallow does not improve, when it is necessary to consider the patient’s underlying disease and general condition, as well as the effects of medications, especially those containing anticholinergics. A previous deprescribing report was associated with improved nutritional intake in older adults, sarcopenic patients with polypharmacy undergoing stroke rehabilitation [28]. As such, deprescribing is another option to improve feeding and swallowing ability.

The present study exhibits several limitations. First, because this is a single-center retrospective study, determining a causal relationship for its results is not possible. Second, details such as swallowing rehabilitation and oral care practices were not considered. Third, drug dosage was not considered. Fourth, we did not consider the onset of sarcopenia dysphagia [8,9]. Furthermore, data on delirium were not collected in this study, and therefore could not be evaluated as a potential confounding factor. Fifth, cumulative past exposure to anticholinergics and its potential effects on swallowing functions were not considered. Finally, we did not consider the effect of anticholinergic drugs other than those indicated by Rudolph et al. [14]. Hence, we cannot rule out the possibility that these issues might have affected the results of this study.

In conclusion, this study showed that the anticholinergic load during hospitalization was associated with the decline of the swallowing function and that the higher the ARS score, the lower the FOIS score is. In the future, examining how much reducing or discontinuing anticholinergics improves the dysfunction in swallowing function will be necessary.

## Figures and Tables

**Table 1:** Baseline of demographic characteristics and laboratory data between ARS (+) group and ARS (–) group.

Characteristic	All Patients (*n* = 542)	ARS (+) (*n* = 164)	ARS (−) (*n* = 378)	*p* Value
Age (y)	79 (73–85)	80.5 (74–86)	79 (73–84)	0.0049 ^†^
Gender *n*, (%)				0.2600 ^§^
Male	201 (37.1)	55 (33.5)	146 (38.6)	
Female	341 (62.9)	109 (66.5)	234 (61.4)	
Length of Stay (d)	65.5 (47–86)	65 (46.3–83)	67 (48–87)	0.2419 ^†^
BW (kg)	50.7 (44.4–59)	49.6 (44.3–58)	51.6 (44.5–59.4)	0.3782 ^†^
BMI (kg/m^2^)	21.9 (19.3–24.5)	21.9 (19.1–24.4)	22 (19.4–24.6)	0.9243 ^†^
Primary diagnosis *n*, (%)				0.4875 ^§^
Cerebral infraction	345 (63.7)	105 (64)	240 (63.5)	
Intracerebral hemorrhage	148 (27.3)	41 (25)	107 (28.3)	
Subarachnoid hemorrhage	49 (9)	18 (11)	31 (8.2)	
Comorbidities *n*, (%)				
Cardiac disease	162 (29.9)	56 (34.2)	106 (28.0)	0.1539 ^§^
Diabetes mellitus	137 (25.3)	42 (25.6)	95 (25.1)	0.9065 ^§^
Hypertension	333 (61.4)	99 (60.4)	234 (61.9)	0.7353 ^§^
Alzheimer’s disease	40 (7.4)	17 (10.4)	23 (6.1)	0.0799 ^§^
Parkinson’s disease	21 (3.9)	18 (11)	3 (0.8)	<0.0001 ^§^
No. of drugs prescribed	3 (1–6)	2 (0–4)	3 (1–6)	<0.0001 ^†^
FIM score (points)				
FIM–T	78 (57–97)	65 (46–91.8)	80 (64–99)	<0.0001 ^†^
FIM–M	53.5 (39–67)	46.5 (28.3–65)	57 (42–67)	0.0002 ^†^
FIM–C	25 (18–31)	22 (14–29)	25 (19–31)	0.0003 ^†^
FOIS score (points)	6 (5–7)	6 (5–7)	6 (5–7)	0.0592 ^†^
Clinical laboratory data				
GNRI	96.4 (83.7–104.5)	93.9 (85.7–103.5)	97.2 (80.9–104.8)	0.7721 ^†^
CRP (mg/dL)	0.2 (0.1–1.7)	0.4 (0.1–2.6)	0.2 (0.1–1.1)	0.0067 ^†^

Abbreviations: ARS—anticholinergic risk scale; BMI—body mass index, BW—body weight; CRP—c-reactive protein; FIM–T—functional independence measure–total; FIM-M—functional independence measure–motor; FIM-C—functional independence measure–cognitive; FOIS—functional oral intake scale; GNRI—geriatric nutrition risk index ^†^: Mann–Whitney U-test; ^§^: Chi-square test.

**Table 2:** Univariate analysis of outcomes between ARS (+) and ARS (–) groups.

Characteristic	All Patients (*n* = 542)	ARS (+) (*n* = 164)	ARS (−) (*n* = 378)	*p* Value
FOIS score at discharge	6 (5–7)	5.5 (4–7)	6 (5–7)	<0.0001 ^†^
Change in FOIS score	0 (−1–1)	0 (−2–1)	0 (−1–1)	0.0070 ^†^

Abbreviations: ARS—anticholinergic risk scale; FOIS—functional oral intake scale. ^†^: Mann–Whitney U-test.

**Table 3:** Multiple linear regression analysis for FOIS score at discharge.

Variable	β	SE	95% CI of β		VIF	*p* Value
Age	−0.008	0.010	−0.029	0.014	1.261	0.3972
Gender (Male)	−0.065	0.072	−0.207	0.077	1.091	0.3699
BMI	0.052	0.024	0.004	0.100	2.195	0.0356
Alzheimer’s disease	−0.067	0.128	−0.319	0.185	1.074	0.6001
Parkinson’s disease	0.121	0.180	−0.232	0.475	1.078	0.5013
Cerebral infarction	0.072	0.104	−0.132	0.276	1.048	0.4868
Intracerebral hemorrhage	0.139	0.118	−0.093	0.372	1.021	0.2402
Subarachnoid hemorrhage	Reference					
Change in the ARS score	−0.118	0.049	−0.214	−0.022	1.093	0.0164
No. of drugs prescribed	−0.007	0.022	−0.043	0.043	1.060	0.9973
FIM–T score	0.018	0.003	0.012	0.024	1.239	<0.0001
CRP	0.003	0.018	−0.033	0.040	1.133	0.8764
GNRI	−0.005	0.008	−0.020	0.012	2.453	0.5793
Constant	4.258	1.133	2.031	6.485		0.0002

Abbreviations: ARS—anticholinergic risk scale, BMI—body mass index, CI—confidence interval, CRP—C-reactive protein, FIM–T—functional independence measure−total, FOIS—functional oral intake scale, GNRI—geriatric nutrition risk index, SE—standard error, VIF—variance inflation factor—R^2^ = 0.146, *p* ≤ 0.0001.

**Table 4:** Subgroup analysis of main outcome.

Variable	FOIS Score at Discharge	*p* Value
Gender		
Male	6 (4–7)	0.3335 ^†^
Female	6 (5–7)	
Stroke subtype		
Cerebral infarction	6 (5–7)	0.2929 ^§^
Intracerebral hemorrhage	6 (5–7)	
Subarachnoid hemorrhage	6 (4–7)	
Change in the ARS score		
−1 point or more decrease ^a^	7 (6–7)	0.0002 ^§^
0 point ^b^	6 (5–7)	
1 point increase ^c^	6 (4–7)	
2 points or more increase ^d^	5 (4–6)	

Abbreviations: ARS—anticholinergic risk scale, FOIS—functional oral intake scale. ^†^: Mann–Whitney U-test, ^§^: Kruskal–Wallis test. Each category of changes in ARS score was performed using the Steel–Dwass test. ^a^ vs. ^b^: *p* = 0.6599, ^a^ vs. ^c^: *p* = 0.2652, ^a^ vs. ^d^: *p* = 0.2229, ^b^ vs. ^c^: *p* = 0.0207, ^b^ vs. ^d^: *p* = 0.0021, ^c^ vs. ^d^: *p* = 0.9890.

## Data Availability

The data are not publicly available owing to opt-out restrictions. Data sharing is not applicable.
